# How Shift Work Impacts on Eating Behaviours Among Nurses: A Cross‐Sectional Study

**DOI:** 10.1002/nop2.70884

**Published:** 2026-09-30

**Authors:** Yun‐Chen Chang, Rocco Mea, Elsa Vitale

**Affiliations:** ^1^ School of Nursing China Medical University Taichung Taiwan; ^2^ Nursing Department China Medical University Hospital Taichung Taiwan; ^3^ San Carlo Hospital Potenza Italy; ^4^ Directorate of Health Professions and Nursing ASL Bari Bari Italy

**Keywords:** behaviour, eating, nurse, shift work

## Abstract

**Aim:**

To evaluate differences in eating behaviours between nurses working exclusively daytime shifts and those engaged in night shift work.

**Background:**

A growing body of evidence suggests that night shift work exposed nurses to an increased risk of chronic conditions, including diabetes, metabolic syndrome, cardiovascular disease and cancer.

**Design:**

An online cross‐sectional study was conducted in May 2022.

**Methods:**

Nurses were recruited through their social media pages on Facebook and Instagram.

**Results:**

A total of 408 Italian nurses participated in the study. Of these, 26.7% worked exclusively daytime shifts (morning and afternoon), while 73.3% also worked night shifts. The majority of participants in both groups were younger than 40 years. Nurses working night shifts more frequently reported shorter professional experience, with < 5 years (daily: 8.1%; night: 25.7%) and < 10 years (daily: 2.2%; night: 21.3%) of employment (*p* < 0.001) and were more often employed on a full‐time basis (*p* = 0.004). In contrast, a significant difference emerged in snack consumption between main meals (*p* = 0.002), with nurses working night shifts reporting a higher frequency of snacking (49.8%) compared with those working exclusively daytime shifts (22.3%).

**Implications for the Profession and/or Patient Care:**

Nurses working daytime shifts reported higher snack consumption compared with their night shift counterparts. Further research will be necessary to examine the nutritional composition of snacks and overall meal frequency.

**Patient or Public Contribution:**

No patient or public contribution.

## 1 Introduction

Nursing staff represented the largest professional group within the healthcare workforce worldwide (World Health Organization 2021; Vitale et al. 2021). Nurses have always been presented a central role in bedside care, clinical monitoring, disease prevention and health promotion. Consequently, the health status of the nursing workforce represented a critical factor in maintaining effective healthcare system performance (Swanson et al. 2020). This concern was particularly relevant in the context of night shift work, which was often characterized by high workloads and conditions that made adherence to a healthy lifestyle challenging (Perry et al. 2015).

Understanding poor health among nurses was essential, as nurses' wellbeing was closely associated with patient outcomes, perceived quality of care and organizational performance (Priano et al. 2018; Brborović et al. 2017). Moreover, suboptimal health among nurses could adversely affect their quality of life and increase the risk of morbidity and mortality (Priano et al. 2018). A growing body of evidence indicated that night shift work exposed nurses to an increased risk of chronic conditions, including diabetes, metabolic syndrome, cardiovascular disease and cancer (Streng et al. 2022). Night shift nurses also appeared to be more susceptible to sleep disorders (Park et al. 2023) and obesity (Klyve et al. 2023) compared with daytime nurses, largely due to circadian rhythm disruption (Brborović et al. 2017; Park et al. 2023; Perry et al. 2015; Streng et al. 2022). These alterations have been associated with differences in caloric and macronutrient intake (Brborović et al. 2017; Klyve et al. 2023; Hulsegge et al. 2016; Gupta et al. 2019; Gioia et al. 2022), leading to irregular meal frequency and suboptimal dietary patterns (Souza et al. 2019).

van Drongelen et al. (2011) investigated the relationship between night shift work and body weight gain, demonstrating a direct association between shift work and increased body weight, findings that have been supported by subsequent studies (Li et al. 2021). However, much of the existing evidence relied on self‐reported data, warranting cautious interpretation of results (Kdekian et al. 2020; Li et al. 2021; Poulsen et al. 2014).

Research focusing on eating behaviours, meal patterns and dietary quality was particularly relevant in the context of nursing shift work, as these factors could influence digestion, metabolism and circadian regulation among night shift nurses (Bonham et al. 2018; Kaneko et al. 2021). Current dietary recommendations emphasized the avoidance of unhealthy eating behaviours, such as skipping breakfast, consuming dinner immediately before bedtime, and eating snacks late at night, as well as limiting high‐fat and sugar‐rich foods, which could contribute to postprandial physiological dysregulation and increased cardiovascular risk (Dimina et al. 2022; Yano et al. 2018). Additionally, meal skipping (Chawla et al. 2021; St‐Onge et al. 2017) and increased consumption of sugar‐sweetened beverages (Green and Syn 2019) have been linked to metabolic dysfunction and cardiovascular disease (Cain et al. 2015; Yu et al. 2016).

Previous studies suggested that night shift work was frequently associated with unhealthy dietary behaviours (Fradkin et al. 2019; Hemiö et al. 2020; Jayedi et al. 2020; Lin et al. 2019; Ross et al. 2019; Souza et al. 2019), characterized by higher overall energy intake (Almoraie et al. 2021; Murakami and Livingstone 2016), increased consumption of energy‐dense foods and beverages (Fradkin et al. 2019; Souza et al. 2019), and more frequent snacking (Hemiö et al. 2020; Ross et al. 2019). These dietary patterns often included higher intakes of carbohydrates (Murakami and Livingstone 2016) and saturated fats (Jayedi et al. 2020; Lin et al. 2019). Night shift nurses commonly reported meal skipping, late dinners, irregular eating schedules and poorer overall diet quality compared with their daytime counterparts (Fradkin et al. 2019; Horton Dias and Dawson 2020).

Nevertheless, findings remained inconsistent, as sleep deprivation, poor sleep quality and circadian rhythm disruption associated with night shift work were themselves independently linked to increased risks of obesity, type 2 diabetes and atherosclerosis (Reutrakul and Van Cauter 2018; Wijaya et al. 2021). Healthy eating behaviours have been associated with adaptive cognitive and behavioural responses to food intake, whereas inflexibility in eating behaviour has been linked to disordered eating patterns (Dahlgren et al. 2019; Zastrow et al. 2009). However, this dimension has not yet been adequately explored among nurses.

Therefore, the present study aimed to assess differences in eating behaviours between daytime and night shift nurses, with a specific focus on meal and snack consumption patterns and flexibility in eating behaviour.

## 2 Materials and Methods

### 2.1 Study Design

An online cross‐sectional study was performed.

### 2.2 Setting

The study was carried out during May 2022. An‐online questionnaire was created full fill the Google Moduli function and spread through Facebook and Instagram ‘Nurseallface’ social pages by inviting nurses to full fill the questionnaire.

### 2.3 Participants

All Italian nurses, who were employed in different care settings, were potentially enrolled in the present study.

### 2.4 Variables

The questionnaire administered investigated the following variables:

- Sex, female or male;
- Age, divided into different age‐groups: under 30 years, 31–40 years, 41–50 years, 51–60 years and over 61 years;
- Years of work experience, divided into main subgroups: < 5 years, 6–10 years, 11–15 years, 16–20 years, 21–25 years, 26–30 years and over 31 years;
- Shift work per day, as the nursing work occurred only during the morning and/or the afternoon (8 AM–8 PM), or also during the night (8 PM–8 AM);
- Nursing educational level, according to the Italian university orientation: until 3 years, from 4 to 5 years and over 5 years;
- Smoking habits: yes or no;
- Weight, expressed in kilograms (kg) and height expressed in meters (m), and then, the BMI value was assessed as meters divided by the height squared. Each BMI value was classified according to the range groups explained in the current literature (Stubbs et al. 2013), as: for BMI values below 18.49, an underweight condition was classified; for values between 18.50 and 24.99, a normal weight condition was assessed; for BMI scores between 25 and 29.99, an overweight condition was identified; and then, for values over 30, an obesity condition was recognized.
- Meal intakes per day: 3 meals/day, 4–5 meals/day, over 5 meals/day;
- Snack intakes among principal meal intakes: yes or no;
- Regular meal assumptions: yes or no;
- Eating flexibility attitude, thanks to the Eating Disorder Flexibility Index (EDFLIX) questionnaire (Dahlgren et al. 2019).

### 2.5 Quantitative Variables

Sex, age, years of work experience, shift work per day, nursing educational level, smoking habits, BMI groups, eating habits in snack intakes and regular or not meal assumptions were considered as categorical variables within specific ranges of assessment abovementioned. Specifically, for variables ‘snack intakes among principal meal intakes’ and ‘regular meal assumptions’ we referred to the main dietary Italian recommendations (Rossi et al. 2022).

As regards the Eating Disorder Flexibility Index (EDFLIX) (Dahlgren et al. 2019), a total of 36 items were proposed assessing Eating Disorders (ED) in specific and general cognitive‐behavioural flexibility and including three sub dimensions, such as: general flexibility (EDFLIX‐GF), food and exercise flexibility (EDFLIX‐FoEx) and weight and shape flexibility (EDFLIX‐WeSh). For each item a Linkert scale was associated from 1 as ‘Strongly disagree’ to 6 as ‘Strongly agree’. Considering reverse item scoring (items no. 02, 05, 06, 07, 08, 09, 10, 12, 13, 14, 17, 18, 25, 26, 27, 28, 29, 34, 35). By summing all the items and only the selected items for each specific sub dimension, a value was obtained by referring higher values indicated higher flexibility to eat capability. The EDFLIX questionnaire showed strong psychometric properties with excellent internal consistency and good construct validity (Dahlgren et al. 2019). In this study, the EDFLIX questionnaire showed a good internal construct validity (*α*_EDFLIX‐tot_ = 0.752).

Since there was a lack of a validated Italian EDFLIX version, the authors firstly proceeded to translate into Italian the 36 total items and then, 30 nurses completed the ‘survey instrument validation rating scale’ (Oducado 2020) in order to better assess if the items were understandable or not. The validation checklist covered a total of 13 criteria in which participants gave a judgement of agreement or disagreement corresponding to the best degree related to the reality experiences. A Likert scale was associated ranging from 1 as ‘strongly disagree’ to 5 as ‘strongly agree’. Criteria included the relevance of the objectives of the study, the depth of the instrument to constructs being assessed, the limits stated, the clarity, the responses stated in a clear way and appropriate to the context proposed, the layout or format of the instrument, the range of answer proposed, the length and the interest of the questionnaire which should reflect the reality experienced by responses.

All nurses answered as ‘strongly agree’ in their validation assessment. Additionally, the items proposed recorded a good validity as *α* = 0.749.

### 2.6 Statistical Methods

All data collected were stored in an Excel data sheet and further processed. All the variables collected were considered as categorical variables and presented as frequencies and percentages, with the exception of the EDFLIX values, which were considered as continuous variables and presented as means ± standard deviations. A multivariate analysis was performed to assess any differences for all categorical variables according to shift typology, as daily or night. Comparisons between day‐shift and night‐shift nurses were assessed thanks to one‐way analysis of variance (ANOVA), since to compare two independent groups, one‐way ANOVA was mathematically equivalent to the independent‐samples *t*‐test having identical statistical inferences. All *p*‐values < 0.05 were considered as statistically significant.

### 2.7 Ethical Concern

In the first part of the questionnaire, there was a clear explanation note of the research study and its relating aim. Each participant gave consent for participation and then completed the questionnaire. All participants who did not give their consent were not enrolled in the study.

Participation was voluntary and anonymous and no individually identifiable information was collected. Questionnaire answers were collected anonymously. Data were analysed and reported in aggregate form to ensure participant confidentiality, in accordance with institutional ethical standards and applicable data protection regulations. Since our questionnaire was spread in online mode thanks to Google Forms, we did not collect any IP addresses.

The present study represented a secondary analysis of data collected from the same cohort previously investigated by Vitale (2022a, 2022b). While the previous study examined work conditions and related risk factors among Italian nurses, the present analysis focused specifically on differences in eating behaviours according to work shift (daytime vs. night shift work). The use of the dataset was covered by the general ethical approval obtained for the original study with protocol no. 0040/56/02/05/2022.

## 3 Results

A total of 408 Italian nurses participated in the study. Among them, 109 (26.7%) were employed exclusively in daytime shifts, while 299 (73.3%) also worked night shifts. The unbalanced group sizes reflected the characteristics of the study sample rather than differential allocation or sampling. The majority of participants were female (75.5%), younger than 30 years of age (36.0%), had < 5 years of professional experience (33.8%), held a basic nursing qualification (75.5%), and were employed on a full‐time basis (91.4%). Most respondents were non‐smokers (58.8%) and had a normal body mass index (57.9%).

When comparing daytime and night shift nurses, statistically significant differences emerged in work‐related characteristics. In both groups, a higher proportion of nurses were younger than 30 years; however, night shift nurses more frequently reported shorter professional experience, with < 5 years (daytime: 8.1%; night shift: 27.9%) and < 10 years (daytime: 25.7%; night shift: 25.0%) of employment (*p* < 0.001). Additionally, night shift nurses were more likely to be employed under a full‐time work regimen compared with daytime nurses (*p* = 0.004) (Table 1).

**TABLE 1:** Sampling characteristics according to shift (*n* = 408).

Sampling characteristics	Daily shift (109; 26.7%)	Daily and night shift (299; 73.3%)	*p*
Sex			
Female	84 (92)	224 (75)	0.698
Male	25 (8)	75 (25)	
Age			
Up to 30 years	33 (30)	114 (38)	0.175
31–40 years	35 (32)	102 (34)	
41–50 years	28 (26)	55 (18)	
51–60 years	13 (12)	24 (8)	
≥ 61 years	0 (0)	4 (1)	
Work experience			
Until 5 years	33 (30)	105 (35)	> 0.001*
6–10 years	9 (8)	87 (29)	
11–15 years	18 (16)	42 (14)	
16–20 years	13 (12)	27 (9)	
21–25 years	12 (11)	12 (6)	
26–30 years	9 (8)	15 (5)	
Over 31 years	15 (14)	11 (4)	
Nursing educational level			
Up to 3 years	82 (75)	226 (76)	0.503
4–5 years	15 (14)	31 (10)	
Over 5 years	12 (11)	42 (14)	
Occupation			
Full‐time	92 (84)	281 (94)	0.004*
Part‐time	17 (16)	18 (6)	
Smoking habit			
Yes	36 (33)	132 (44)	0.053
No	73 (67)	167 (66)	
BMI values			
≤ 18.49: Underweight	6 (6)	13 (4)	0.125
18.50–24.99: Normal weight	59 (54)	177 (59)	
25.00–29.99: Overweight	37 (34)	69 (23)	
≥ 30: Obese	7 (6)	40 (14)	

* *p* < 0.05 is statistically significant.

When eating patterns were examined (Table 2), no statistically significant differences were observed between daytime and night shift nurses with respect to the number of meals consumed per day (*p* = 0.122) or the regularity of meal consumption (*p* = 0.059). In contrast, a significant difference emerged in snack consumption between main meals (*p* = 0.002), since nurses working exclusively daytime shifts reported a higher frequency of snacking (83%) compared with nurses employed during night shift (68%).

**TABLE 2:** Eating patterns among nurses enrolled according to shift (*n* = 408).

Sampling characteristics	Shift		*F*	*p*
	Daily (109; 26.7%)	Daily and night (299; 73.3%)		
Meals intake per day				
Until 3 meals/day	50 (46)	117 (39)	2.405	0.122
3–4 meals/day	50 (46)	143 (48)		
Over 5 meals/day	9 (8)	39 (13)		
Eating between principal meals				
Yes	91 (83)	203 (68)	9.832	0.002*
No	18 (7)	96 (32)		
Regular meals intakes				
Yes	68 (62)	155 (52)	3.599	0.059
No	41 (38)	144 (48)		

* *p* < 0.05 is statistically significant.

Finally, analysis of the EDFLIX total score and its associated sub dimensions revealed no statistically significant differences between daytime and night shift nurses. These findings suggest that flexibility in eating behaviours is independent of nursing shift work (Table 3).

**TABLE 3:** Flexibility in eating habits among nurses enrolled according to shift (*n* = 408).

EDFLIX score/shift	*μ* ± SD	95% CI Min.–Max.	*F*	*p*
EDFLIX total				
Daily (109; 26.7%)	139.4037 ± 18.40296	135.9097–142.8976	1.915	0.167
Daily and night (299; 73.3%)	142.1405 ± 17.40343	140.1598–144.1212		
EDFLIX‐GF				
Daily (109; 26.7%)	62.9908 ± 12.81961	60.5569–65.4247	2.555	0.111
Daily and night (299; 73.3%)	65.0401 ± 10.92370	63.7969–66.2834		
EDFLIX‐FoEx				
Daily (109; 26.7%)	50.4220 ± 7.57101	48.9846–51.8594	2.699	0.101
Daily and night (299; 73.3%)	51.8027 ± 7.48900	50.9504–52.6550		
EDFLIX‐WeSh				
Daily (109; 26.7%)	25.9908 ± 5.64292	24.9195–27.0622	0.856	0.356
Daily and night (299; 73.3%)	25.2977 ± 7.04123	24.4963–26.0990		

Abbreviations: *μ* ± SD, mean ± standard deviation; CI, confidence interval; EDFLIX‐GF, general flexibility; EDFLIX‐FoEx, food and exercise flexibility; EDFLIX‐WeSh, weight and shape flexibility.

* *p* < 0.05 is statistical significant.

## 4 Discussion

The present study aimed to investigate differences in eating behaviours between daytime and night shift nurses, with a particular focus on meal and snack consumption patterns and flexibility in eating behaviour.

Most participants were employed in night shift work (73.3%) and were relatively young, with a large proportion aged under 30 years in both groups. In addition, nurses working night shifts more frequently reported shorter professional experience, with < 5 years (daytime: 8.1%; night shift: 25.7%) and < 10 years (daytime: 2.2%; night shift: 21.3%) of employment (*p* < 0.001). No statistically significant differences were observed between daytime and night shift nurses with respect to smoking status (*p* = 0.053) or body mass index (BMI) categories (*p* = 0.125). Considerable attention has been directed towards the potential impact of night shift work on overweight and obesity; however, the evidence remained inconsistent. While several studies have reported a positive association between night shift work and overweight or obesity (Balieiro et al. 2014; Chaput et al. 2023; Givens et al. 2015), others have failed to confirm this relationship (Peplonska et al. 2015; Yoon et al. 2016), findings that were consistent with the results of the present study. Similarly, the absence of an association between smoking habits and night shift work aligns with previous findings reported by Samhat et al. (2020).

In contrast, a significant difference emerged in snack consumption between main meals (*p* = 0.002), since nurses working exclusively daytime shifts reported a higher frequency of snacking (83%) compared with those working night shifts (68%). These findings were in disagreement with evidence from Lebanese nurses, which demonstrated that night shift work was associated with less healthy eating behaviours, irregular meal timing and increased consumption of high‐fat and sugar‐rich snacks during night shifts (Samhat et al. 2020). Such patterns could contribute to an increase in BMI over the course of nurses' careers, potentially explaining higher BMI values observed among older nurses compared with younger counterparts. Nevertheless, in the present study, BMI did not differ significantly between daytime and night shift nurses, despite substantial evidence suggesting that night shift work contributes to weight gain and increases in BMI and waist circumference, likely due to excessive caloric intake during night work (Bonnell et al. 2017; Griep et al. 2014; Zhang et al. 2020).

No significant differences were observed between the two groups in terms of the number of meals consumed per day (*p* = 0.286) or regularity of meal intake (*p* = 0.072). These findings were in disagreement with some previous studies (Broussard et al. 2016; Zhu et al. 2019). Thus, higher snacking frequency among night shift nurses could be explained by physiological mechanisms, such as alterations in appetite‐regulating hormones, including reduced leptin and increased ghrelin levels, which could disrupt circadian rhythms and influence food choices, leading to increased snacking and meal skipping (James et al. 2017; Lin et al. 2023). This pattern has been well described in the review conducted by Souza et al. (2019), which reported that night shift nurses frequently skipped breakfast and lunch accompanied by increasing snack consumption during night shifts. However, despite these trends, the present study did not identify significant differences in overall meal frequency or regularity between groups.

Finally, analysis of the EDFLIX total score and its related sub dimensions revealed no significant differences between daytime and night shift nurses. These results could suggest that flexibility in eating, exercise and body shape–related behaviours was not influenced by night shift work. This finding was in disagreement with previous literature reporting reduced levels of physical activity among night shift nurses due to fatigue associated with night work, often accompanied by increases in BMI (Lisco et al. 2024; Ulhôa et al. 2015; Vitale 2022a). On the other hand, the present study did not identify significant differences in these behavioural domains between groups. Consequently, further well‐designed studies are warranted to more precisely identify the factors through which night shift work may influence health‐related behaviours among nurses.

### 4.1 Limitations

The present study presented several limitations that should be acknowledged. First of all, the recruitment strategy through social media pages (Facebook and Instagram) was convenient but introduced substantial selection bias and the lack of generalizability of our findings despite the representativeness of our sample. Additionally, the distribution between the two groups was unbalanced, with a substantially smaller number of nurses working exclusively daytime shifts compared with those working night shifts without considering fixed/set shifts and rotating shift schedules within the night group, as these types of schedules could likely influence eating behaviours based on predictability/changing schedules. Secondly, the number of items assessing meal intake was limited, and the quality of the information collected could have been influenced by the use of an online data collection method. Finally, the study employed a cross‐sectional design rather than a correlational or experimental approach. Consequently, while differences between daytime and night shift nurses were carefully examined, no causal inferences could be drawn regarding the relationship between shift work, dietary behaviours, body weight or physical activity. Therefore, non‐significant findings should be interpreted with appropriate caution.

## 5 Conclusion

The present study demonstrated that nurses working daytime shifts reported a higher frequency of snacking compared with their night shift counterparts. No further significant differences were identified between daytime and night shift groups considering body mass index, smoking habits, physical activity or flexibility in eating behaviour. Nevertheless, further research will examine the nutritional composition of snacks and explore meal patterns in greater depth, moving beyond the simple assessment of the number of meals consumed per day. This study could be considered a preliminary contribution to a complex and multifaceted topic, as night shift nurses appeared to experience feature alterations in their relationship with food and physical activity. However, consistent with existing literature, the identification of key underlying factors remained highly controversial.

According to our findings, the implementation of targeted health promotion strategies aimed at fostering healthy eating behaviours among nurses could be essential to support their health and wellbeing and to prevent long‐term adverse health outcomes.

## Author Contributions

**Yun‐Chen Chang:** visualization, supervision, funding acquisition. **Rocco Mea:** investigation, methodology, data curation. **Elsa Vitale:** writing – review and editing, writing – original draft, conceptualization, supervision, visualization, validation.

## Funding

This work was supported by the National Science and Technology Council, Taiwan (grant no. NSTC 115‐2410‐H‐039‐010) and by China Medical University (grant no. CMU115‐AWARD‐01). The funders had no role in the design of the study, in the collection, analysis or interpretation of data, in the writing of the manuscript, or in the decision to submit it for publication.

## Ethics Statement

The study had ethics approval from the Ethical Committee of Polyclinic in Bari, Italy, with protocol no. 0040/56/02/05/2022. The research was conducted in accordance to Declaration of Helsinki. During data analysis, no individual could be individually identified.

## Consent

All participants were over 18 years of age, were provided with a Participant Consent and Information Sheet and informed consent was obtained from all participants. Each participant gave their own consent to participate in the present study.

## Conflicts of Interest

The authors declare no conflicts of interest.

## Data Availability

The data that support the findings of this study are available from the corresponding author upon reasonable request.
